# Graduated compression stockings in hip fractures

**DOI:** 10.1308/003588412X13171221592492

**Published:** 2012-10

**Authors:** A Alsawadi, M Loeffler

**Affiliations:** Colchester Hospital University NHS Foundation Trust,UK

**Keywords:** Hip fracture, Thromboprophylaxis, Graduated compression stocking

## Abstract

**INTRODUCTION:**

Hip fractures are the most common cause of acute admissions to orthopaedics and in the UK approximately 70,000–75,000 hip fractures occur annually. Hip fractures carry a significant risk of developing a venous thromboembolism. The National Institute for Health and Clinical Excellence (NICE) estimated that the risk of developing a venous thromboembolism in patients with hip fractures who do not receive thromboprophylaxis is 43%. In their recent guidelines, NICE recommended that combined mechanical and pharmacological thromboprophylaxis should be offered to patients undergoing hip fracture surgery and mechanical prophylaxis should be commenced at admission. The aim of this review was to search for available evidence that could support using graduated compression stockings combined with low molecular weight heparin (LMWH) in hip fracture patients.

**METHODS:**

NICE guidelines and the reference list of the guidance were reviewed and a thorough literature search was performed on main electronic databases (MEDLINE®, Embase™ and the Cochrane Library).

**RESULTS:**

A literature search was unable to find sufficient evidence to support the use of graduated compression stockings combined with LMWH in hip fracture settings. The guidelines are critically reviewed and the available evidence discussed.

**CONCLUSIONS:**

The evidence supporting these recommendations is very limited and there is considerable concern regarding the safety and efficacy of the mechanical devices used in thromboprophylaxis. Further studies are needed urgently before specific guidelines can be agreed confidently for patients with hip fractures.

Approximately 70,000–75,000 hip fractures occur annually in the UK and they are the most common cause of acute admission to orthopaedics units.^1^ Hip fractures carry a significant risk of developing a venous thromboembolism (VTE). The National Institute for Health and Clinical Excellence (NICE) estimated that the risk of developing a VTE in patients with fractures of the proximal femur not receiving thromboprophylaxis is 43% (37% risk of deep vein thrombosis [DVT] and 6% risk of pulmonary embolism) compared with the risk of major bleeding events in the same population, which is 2%.^2^ Geerts *et al* estimated the absolute risk of DVT (based on objective diagnostic testing for DVT) in hip fracture patients without VTE prophylaxis to be 40–60%^3^ and this can be reduced to as low as 1.6% through the use of thromboprophylaxis.^4^

Agreed guidelines were published by NICE in January 2010 for VTE prophylaxis in hospitalised patients and these focused on several patient groups including those with hip fractures.^2^ Randomised controlled trials (RCTs) and systematic reviews were consulted and several meta-analyses were conducted. However, specific evidence for hip fracture patients was lacking within the NICE guidelines.^2^ Some of the recommendations for hip fracture patients were generalised from elective arthroplasty patients although the differences between the two patients groups are considerable. In addition, hip fracture patients often have coexisting medical problems that contraindicate the use of certain mechanical or pharmacological thromboprophylaxis and the risk of major complications must be considered carefully before their use.^5^

In the published guidelines, NICE recommended that combined mechanical (graduated compression stocking, foot impulse devices or intermittent pneumatic compression devices) and pharmacological (fondaparinux sodium, low molecular weight heparin [LMWH] or unfractionated heparin) prophylaxis should be offered to all patients undergoing hip fracture surgery and mechanical VTE prophylaxis should be commenced at admission.^2^ Mechanical VTE prophylaxis should be based on individual patient factors and could be any of the three following options: antiembolism stockings (AES)/graduated compression stockings (GCS), foot impulse devices (FID) or intermittent pneumatic compression devices (IPCD).

From a practical point of view, FID and IPCD can be classified as one group while AES and GCS constitute a second. IPCD and FID use an ‘active’ mechanism whereas GCS and AES use a ‘passive’ method.^2^ In the NICE guidelines (and in this review), the acronym GCS is used to refer to both AES and GCS.

From our own observations, in practice, GCS are used more commonly than IPCD/FID. A literature search did not reveal any statistical evidence to support or disprove this although Cohen *et al* estimated the use of GCS to be 70% for UK patients.^6^ Rajaganeshan *et al* conducted a national survey in the UK to determine the use of thromboprophylaxis in hip fracture patients.^7^ A questionnaire was sent to 1,648 orthopaedic consultants and resulted in a 44% (*n*=723) response rate. Of those who responded, 320 (58%) used mechanical prophylaxis (mechanical prophylaxis only or combined with pharmacological prophylaxis), 11 (3%) used stockings, 30 (9%) used foot/ankle pumps and 60 (19%) used Flowtron® boots (ArjoHuntleigh, Luton, UK). The majority (96%, *n*=219) reported the use of mechanical devices combined with other prophylactic methods but did not provide further clarification on what combinations were used. In addition, the response rate in this study was low and it was conducted more than four years prior to the NICE guidelines. It does not therefore reflect current practices.

Another reason to think that IPCD/FID are used less commonly than GCS is that they raise concerns regarding compliance and this has been reported to be a major issue.^5,8^ This is likely to be more problematic in the hip fracture population.

Based on this background and due to this lack of evidence on best practice for VTE prophylaxis in hip fracture patients, this review focused on this patient group. The primary aim was to search for evidence to support the use of GCS in conjunction with LMWH in hip fracture patients. The review also examined NICE guidelines and the general available evidence for the use of GCS in hip fracture patients.

## Methods

The NICE VTE guidelines^2^ were reviewed for available evidence on which the recommendations were based. The reference list of the NICE guidelines was also searched for relevant studies. A thorough literature search on the subject was undertaken in main electronic databases including MEDLINE®, Embase™ and the Cochrane Library. The keywords searched were ‘hip fracture’, ‘thromboprophylaxis’, ‘thromboembolism’, ‘pulmonary embolism’, ‘deep vein thrombosis’, ‘mechanical prophylaxis’ and ‘graduated compression stockings’. MeSH (Medical Subject Headings) terms were also searched. The search strategy was not limited to time of publication or type of article but only papers written in English were sought.

## Results

The literature search did not identify any studies with a focus on hip fracture patients only comparing LMWH alone with combined LMWH and GCS. However, one study was mentioned in the NICE guidelines that compared hip fracture patients who received fondaparinux with those who received fondaparinux plus GCS.^6^ In the discussion below, the focus is on the NICE guidelines and the available data on which the guidance are based.

## Discussion

### Mechanical prophylaxis and hip fracture patients

NICE identified 30 RCTs that reported at least one of the three main outcomes (DVT, pulmonary embolism and major bleeding).^2^ Some of these RCTs investigated more than two modalities of thromboprophylaxis. The data from most of these RCTs had been extracted from systematic reviews and, where applicable, the study was cited in the evidence table for that review. RCTs covering patients with hip fractures were included in six of the systematic reviews although two studies included a mixed group of patients with both hip fractures and elective hip replacements. Of the 30 RCTs, 23 were included in the network meta-analysis for DVT.

The quality of the included studies was evaluated and the included RCTs were either appraised individually or retrieved from systematic reviews that in turn had been appraised.^2^ However, 78% of the 23 RCTs included in the meta-analysis were published before 1990. Consequently, some of the surgical techniques cited are no longer in current practice. In addition, 61% of the included RCTs had fewer than 100 patients and, taken together, these factors may severely limit the available evidence.

In the section of the guidelines entitled *Summary of Evidence for Mechanical and Pharmacological Prophylaxis* (pp148–153), there is no significant difference noted between GCS combined with LMWH and LMWH alone or between GCS combined with fondaparinux and fondaparinux alone, in the outcome of DVT and pulmonary embolism, in all available evidence and across all patients groups (medical, surgical and trauma patients).^2^ GCS are, however, linked to a significant increase in adverse events in stroke patients, such as skin breaks, ulcers, blisters and skin necrosis.^9^

In a multicentre, outcome blinded RCT, Dennis *et al* allocated 2,058 stroke patients, recruited internationally from 64 centres, to routine care plus thigh-length GCS or routine care avoiding the use of GCS.^9^ The results showed that thigh-length GCS did not result in a statistically significant difference in the measured outcome (symptomatic or asymptomatic DVT in the popliteal or femoral veins). In fact, complications (eg ulcers, blisters and skin breaks) were significantly higher in patients allocated to the GCS group even though patients with peripheral vascular disease or diabetes, those who had sensory neuropathy or those for whom the responsible clinician or nurse judged that GCS might cause a skin break were excluded.

NICE argued that these results were found in a special group of patients (stroke patients) and that they are unlikely to be transferrable to other populations.^2^ However, it can also be argued that the safety of GCS in a hip fracture population has not been proven. No similar studies on hip fracture patients were identified by the guidelines or could be found through a literature search. Additionally, none of the 30 RCTs reviewed by NICE that investigated the different methods of thromboprophylaxis compared LMWH with combined mechanical and pharmacological prophylaxis following hip fracture surgery.

Previous randomised studies have reported a significant reduction in post-operative thrombosis associated with compression stockings. Ohlund *et al* conducted a trial of 63 patients who received mixed elective hip surgery^10^ and Fredin *et al* undertook a study of 150 patients admitted for elective total hip arthroplasty.^11^ Both groups in both studies received dextran as their primary thromboprophylaxis. It is very likely that the small sample size contributed significantly to the results of these studies. In addition, the use of dextran is now an outdated intervention for thromboprophylaxis.^2^

In a larger and more recent multicentre randomised trial on the use of GCS in association with hip surgery, 400 patients who received fondaparinux were compared with 395 patients who received fondaparinux plus GCS.^6^ No difference was observed in the prevalence of VTE between the two groups. Despite careful selection, 2% of the patients developed complications related to the use of stockings. Although the study had a large sample size, was well randomised and the level of compliance was high, it is difficult to generalise the results for hip fracture patients. The population of the study was a mix of elective and hip fracture cases with only about 5% having a fractured hip. Hip fracture patients are usually fragile and elderly in comparison with fit arthroplasty patients admitted for surgery electively. In a systematic review of 31 trials, Handoll *et al* did not find any randomised trial testing the use of GCS in hip fracture patients.^8^

In addition to safety concerns regarding GCS, there is some survey evidence that they are associated with reduced quality of life (eg disutility and discomfort).^2,12^ The burden of proof should therefore be on the intervention (ie mechanical prophylaxis) and due to the lack of evidence describing their benefit and the presence of concerns regarding their potential harm, it is questionable as to whether applying the guidelines of mechanical prophylaxis to hip fracture patients should be accepted or whether further investigations and studies should be undertaken to provide evidence for their use in improving patient outcomes.

### The use of surrogate endpoints

It has been a routine practice for trials examining the clinical effectiveness of thromboprophylaxis to set an outcome of symptomatic and asymptomatic DVTs, and detection of asymptomatic DVT in most of the research on thromboprophylaxis in orthopaedic surgery has been based on venography.^13^ However, the safety and efficacy of venography in detecting asymptomatic DVT has been widely questioned. First, venography is invasive, uncomfortable and possibly thrombogenic,^13^ and second, studies have shown that anticoagulant prophylaxis may delay the peak onset of DVT.^14–16^ Sikorski *et al* found that the peak onset of DVT in untreated post-total hip replacement patients was on the fourth day, a second smaller incidence peak occurred on day 13 and the risk of DVT was over by day 17.^15^ In contrast, the peak incidence in the group treated with heparin was on day 6 and the risk of thrombosis continued to day 18 or beyond.

A single venogram can only measure prevalence rather than incidence and it will not detect early thrombi or those that occur later. Additionally, repeating venography on several occasions to reduce this discrepancy between prevalence and incidence is impractical.^13^ However, venography does have some advantages. It is simple and easy to perform,^17^ and it has also been argued to be more sensitive than non-invasive methods such as ultrasonography for the diagnosis of asymptomatic DVT.^18^

Ultrasonography has been suggested as an alternative non-invasive and repeatable diagnostic tool.^13,19,20^ Nevertheless, its accuracy and sensitivity have been questioned, especially for detecting asymptomatic DVT.^18,21,22^ Both complete compression ultrasonography and colour-flow Doppler ultrasonography have been trialled for this purpose.^19^ Several studies have compared these two modalities of ultrasonography^22^ or ultrasonography and venography^19,20,23,24^ but all these studies were carried out on elective arthroplasty patients.

A literature search was unable to find sufficient data for similar studies but in a hip fracture setting. Mitra *et al* found no correlation between clinical symptoms and venography findings for post-operative screening in hip fracture patients.^25^ The limitation of this study was clear: a very small number of included patients (*n*=72). Nevertheless, the results are variable and no definitive conclusions could be drawn.

One of the disadvantages of ultrasonography is that it appears to be operator dependent and there are discrepancies between readers.^20,23,26^ These factors have probably contributed to the discrepancies in the findings between the aforementioned studies. In some of the literature, ultrasonography as a diagnostic tool for DVT is considered to be the imaging method of choice for patients with clinically suspected DVT^27^ but its use for post-operative screening for asymptomatic DVT has not been specifically verified.^19^

The timing of screening is another area of disagreement and variable target days have been investigated by different researchers. Ciccone *et al* performed ultrasonography and venography on the fifth to seventh day post-operatively^19^ while in the study by Leutz and Stauffer ultrasonography and venography were performed 3–9 days after surgery^23^ and Schellong *et al* performed venography 5–9 days after surgery and ultrasonography within 24 hours after venography.^20^ This choice of timing is based on convenience rather than epidemiological or haematological evidence since this period is the typical duration of the hospital stay following joint arthroplasty.^13^

The dilemma regarding setting asymptomatic thromboembolism as an outcome does not end at what screening tool should be used and when the screening should be carried out as it is not fully clear whether one or both legs should be imaged. There are very limited clinical data to support the use of bilateral ultrasonography in patients with suspected unilateral DVT.^28^ In post-operative surveillance, some researchers have favoured screening the operated leg only^25^ but many other authors have suggested that bilateral venography is crucial. More recently, Warwick and Samama have reported that up to 20% of post-operative DVT occurs in the contralateral leg.^13^ In a systematic review of prospective studies that gave DVT as the primary outcome based on bilateral venography following surgery for elective hip or knee arthroplasty or hip fracture, the risk of isolated DVT in the non-operated leg was estimated to be 4–5%.^29^ The authors concluded that performing venography on both legs reduced the risk of missing the diagnosis and improved the efficacy of the study.

Previous consensus guidelines have been based on the meta-analysis of large numbers of small trials that have used surrogate endpoints such as venography, ultrasonography and lung scanning,^30,31^ and most published trials are small with the power only to detect radiological differences in DVT.^32^ There is concern regarding the applicability of meta-analysis based on a large number of studies with a small number of patients and whether the results reflect clinically significant events.^31,33,34^

Recording the outcome of asymptomatic DVT is not without its advantages. Asymptomatic thrombi occur much more often than those that are clinically symptomatic. Therefore, surrogate endpoints are used because they provide statistical conclusions that can be reached via a smaller number of patients.^31^ Lee *et al* found that the use of bilateral venography reduces the required sample size by 16–25% compared with ipsilateral venography.^29^

There are significant concerns regarding the findings of these studies and their use in the clinical guidelines published by NICE.^2^ In clinical medicine, physicians and surgeons should be interested in clinical outcomes^13^ and it is a matter for debate whether clinical practice should be based on surrogate endpoint findings.

## Conclusions

In the NICE guidance published in 2010, the use of a combination of mechanical and pharmacological prophylaxis in hip fracture patients was recommended (unless there is contraindication).^2^ The evidence that supports this is very limited and there is considerable concern regarding the safety and efficacy of the mechanical devices of VTE prophylaxis. Researchers should be encouraged to further explore this area, which is lacking in good quality evidence, and it is to be hoped that NICE will consider this issue in the future review of the guidance.
